# The Use of Network Theory for Analyzing Switching Behaviors: Assessing Cognitive and Educational-Based Intervention for Promoting Health

**DOI:** 10.3389/fpsyg.2018.01095

**Published:** 2018-06-26

**Authors:** Giorgio Gronchi

**Affiliations:** Psychology Section, Department of Neuroscience, Psychology, Drug Research and Child's Health, University of Florence, Florence, Italy

**Keywords:** drugs switching, health psychology, nudge, educational interventions, network theory, graph theory

## Introduction

New technologies and the increasing presence of Internet-connected computer systems in every aspect of our lives (e.g., during work, free time, or times dealing with health issues) have made a large amount of information available about human behavior (Barsocchi et al., 2015; Watanabe et al., 2017). This information is systematically stored in database systems. For example, administrative databases contain much information about health-related behaviors and are constantly updated so that every time someone receives a drug by a physician's prescription, that information is recorded in the database (Parri et al., 2015; Giusti et al., 2016, 2017; Giudici et al., 2017; Cianferotti et al., 2018). In this regard, a relevant topic for many psychological applications is *switching behaviors*: data which describe the shift from a certain behavior or situation to the same or another behavior or situation. The phrase *behavior or situation* refers to a variety of health conditions and behaviors (e.g., dietary habits); in this paper, I will consider drug consumption: a type of critical health-related information which can be retrieved from administrative databases. The prescription of a given drug, for example, and the subsequent prescription of the same or another drug (i.e., *no switch* compared to *switch*) may be found in a database. From a methodological point of view, however switching behavior has been usually analyzed by aggregating data in order to compare a group of individuals who have switched in a given period of time to a group of individuals who have not switched during the same period (Ganguli, 2002; Thiebaud et al., 2005; Ideguchi et al., 2008). On one hand, given the complexity of large databases containing drug prescriptions of tens of thousands of people for several years, it is necessary to simplify and adopt some strategy to analyze them in a manageable way. On the other hand, this approach does not allow us to exploit the actual structure of drug-switching behaviors (e.g., answering questions such as “Given a certain drug, what are the other drugs with which a switch is more likely?”). The aim of this paper is to show how graph theory (Kolaczyk and Csárdi, 2014; Luke, 2015) could be employed to model and improve the analysis of switching behavior; indeed, the availability of database information and the potential for a mathematical modeling approach such as network theory—which is still not widely used with this aim—could greatly improve the analysis of switching behavior (in other fields—for example, marketing research—a similar approach has been already proposed, see Iacobucci et al., 1996). The paper is organized as follows: first, it will describe why an improvement in data analysis approaches for investigating adherence and, in particular, switching behaviors is important for the field of psychology. Then, discussion of the benefits of adopting network analysis compared to traditional analysis will follow.

## The need in psychology for deeper investigation of adherence and switching behaviors

When a physician gives a patient instructions about a healthcare program (e.g., prescriptions, dietary habits, and physical activities), the patient may or may not follow medical advice. *Adherence* (also called compliance) measures the degree to which a patient correctly follows the physician's directions, whereas *persistence* refers to taking the treatment for the prescribed duration. From a psychological point of view, this set of complex behaviors involves many variables (e.g., habits, motivation, intentions, and beliefs). Within health psychology several theoretical models have been proposed to design behavior change interventions. Michie et al. (2011) wrote:

“Behavior change interventions can be defined as coordinated sets of activities designed to change specified behavior patterns. In general, these behavior patterns are measured in terms of the prevalence or incidence of particular behaviors in specified populations (e.g., delivery of smoking cessation advice by general practitioners). Interventions are used to promote uptake and optimal use of effective clinical services, and to promote healthy lifestyles.” (p. 1).

More recently, within thinking psychology, the dual process theory of thought (Sloman, 1996) has been exploited to achieve those goals. According to this theory, thought is the result of two forms of thinking: (a) a slow, effortful, deliberative process and (b) a fast, effortless, default- and associative-based process. Exploiting the properties of the latter form of reasoning, the Nudge framework proposed by Thaler and Sunstein (2009) suggests interventions on the architecture of choice using defaults and indirect suggestions to obtain nonforced compliance and to influence the motives and decision-making processes of people. Such “cognitive-based” interventions can be very effective in promoting positive behaviors and increasing the acceptance of physicians' suggestions (Pampaloni et al., 2015).

Accessibility to administrative databases (Parri et al., 2015; Giusti et al., 2016, 2017; Cianferotti et al., 2018) allows for the possibility of systematically measuring health-related behavior, thus making it possible to ascertain if patients have a sufficiently high adherence to prescribed drugs (Cianferotti et al., 2015, 2017; Vannucci et al., 2017a,b). In the case of low compliance, it is desirable to conduct some type of intervention (e.g., classic behavior change intervention or Nudge-based), and thus it would be necessary to monitor the effectiveness of the intervention by analyzing the administrative database.

Drug-switching behavior represents a crucial topic in this regard. In some cases, the physician may ask a patient to switch their medication. In order to increase bone mass, for example, bisphosphonates may be used as a treatment option; in current medical practice patients might be given two or more bisphosphonate drugs in sequence (Ideguchi et al., 2008). Administrative database records provide information about drugs actually taken in the pharmacies, so it is possible to acquire data about how prescriptions of various drugs change in sequence. Such analyses may help to answer important questions like “Do patients switch drugs in the way prescribed by the physician?”; “What switch pattern is associated with the best clinical outcome (e.g., reduction in probability of fracture)?”; and “Do the educational interventions succeed in promoting the desired switch pattern of drugs?”

Another example f drug-switching behavior relates to generic and branded drugs. The physician may prescribe a drug, and when the patient goes to the pharmacy, the patient can choose (according to advice from the pharmacy clerk) to take either the branded or generic drug. In certain cases, however, the two versions of the drug may differ in terms of efficacy or tolerability (Andermann et al., 2007; Kesselheim et al., 2008). Patients with chronic disease may take their drugs for a long time, switching continuously from branded drugs to generic ones. Again, the pattern of switching behavior can be related to clinical outcomes (i.e., health-related variables, such as the probability of fracture or mortality).

## Going beyond traditional analysis: the advantages of using network theory

A researcher who is interested in investigating the topics described in the previous section may obtain data from administrative databases. Information is usually organized in rows containing, on the columns, the following terms about each prescription: (a) the date when the drug was dispensed, (b) the ID of the patient to whom the drug was prescribed, (c) the typology of drug, and (d) other variables. By sorting the data by date, patient ID, and typology, it is possible to know if a patient, in a given interval of time, has switched drugs (or classes of drugs). Alternatively, it is possible to compute how many times a patient has switched.

Traditional analyses are usually limited to the comparison between a group of patients who have not switched drugs versus a group of patients who have switched drugs in a given interval of time (Saag et al., 2002; Ideguchi et al., 2008; Martin et al., 2009). For example, switch versus no switch patients can be compared in terms of commonly employed adherence measures (e.g., medical possession ratio [MPR]). These methods are usually adequate to handle databases with only a few different types of drugs, and they have the advantage of simplicity; however, the main disadvantage is that analyses of databases with these traditional methods can miss the actual complexity of switching behavior, especially when using several different drugs and very large databases. Indeed, with a database of acceptable size, it is possible to construct a matrix reporting the different drugs in rows and columns and the frequency of switches in each cell, and this adjacency matrix is amenable to being analyzed in terms of network theory (Gronchi et al., 2013, 2014; Kolaczyk and Csárdi, 2014; Luke, 2015). Graph theory, in turn, can provide several indices for going beyond traditional approaches. Following this idea, each drug is considered as a node in a graph, and the (possible) switch is viewed as an edge. Among the simplest indices that can be computed on the adjacency matrix is degree centrality (i.e., the number of links incident upon a node). This will allow answers to questions such as “How many different drugs are switched to that particular drug?” Closeness centrality can also be useful: It allows measurement of the degree to which a drug is “near,” in terms of switch, to all other drugs in a network. This index computes the shortest paths between all nodes (in our case, drugs), and then it assigns to each node a score based on its sum of shortest paths. Closeness centrality can thereby help to find a drug to which all the other drugs tend to shift with a few number of switches.

In clinical situations where several types drugs are administered (e.g., osteoporosis research; Ideguchi et al., 2008) or where large databases are used, drug switches may interact in a complex way, forming structured patterns. In this case, network analysis has made available many clustering algorithms (Sales-Pardo et al., 2007; Rosvall and Bergstrom, 2008; Lancichinetti et al., 2011; Gronchi et al., 2013, 2014). By using those algorithms, it is possible to detect how the different switch patterns of drugs form distinct groups. For example, drugs A, B, and C might usually form a cluster (i.e., a set of drugs that usually switch from one to another of the same group), whereas drugs D, E, and F might form a different cluster.

There are some limitations in the use of network theory for analyzing drug-switching behavior. First, gaining the official permissions to access administrative databases at single-prescription and single-patient levels is not easy; even if the IDs are anonymous, these data are sensitive information and they are usually only available to the researcher in aggregate form. This access, however, is necessary to construct the adjacency matrix. Moreover, in order to apply network theory, databases must be large and related to several drugs in order to have a structured adjacency matrix with enough rows and columns. Only in particular clinical contexts, however, is it meaningful to investigate the switch among several drugs because it is necessary to employ network theory.

## Conclusion

This paper advocates for the use of network theory, which can be used to more precisely compare matrices of drug-switching information obtained from different samples (e.g., control versus intervention groups), thus going beyond the simple probability of switch. Systematic and meta-analytic reviews (Borenstein et al., 2009; Tallandini et al., 2014, 2016) regarding drug-switching behavior are necessary for evaluating the feasibility of the use of network theory in this respect. Its expanded use for analyzing switching behavior may allow for better investigations of health-related behavior (e.g., the measure of compliance with physicians' indications) and the efficacy of educational and cognitive-based interventions for enhancing adherence.

## Author contributions

The author confirms being the sole contributor of this work and approved it for publication.

### Conflict of interest statement

The author declares that the research was conducted in the absence of any commercial or financial relationships that could be construed as a potential conflict of interest.
